# Fecal Proteome Profile in Dogs Suffering from Different Hepatobiliary Disorders and Comparison with Controls

**DOI:** 10.3390/ani13142343

**Published:** 2023-07-18

**Authors:** Matteo Cerquetella, Sara Mangiaterra, Francesco Pinnella, Giacomo Rossi, Andrea Marchegiani, Alessandra Gavazza, Evelina Serri, Alessandro Di Cerbo, Carlotta Marini, Daniela Cecconi, Daniela Sorio, Veronica Marchetti, Silvia Vincenzetti

**Affiliations:** 1 School of Biosciences and Veterinary Medicine, University of Camerino, Via Circonvallazione 93/95, 62024 Matelica, MC, Italy; 2 Futuravet Veterinary Referral Center, 62029 Tolentino, MC, Italy; 3 Department of Biotechnology, University of Verona, Strada le Grazie 15, 37134 Verona, VR, Italy; 4 Centre for Technological Platforms (CPT), University of Verona, Piazzale L.A. Scuro 10, 37134 Verona, VR, Italy; 5 Department of Veterinary Sciences, University of Pisa, Via Livornese, San Piero a Grado, 56122 Pisa, PI, Italy

**Keywords:** dog, fecal proteomics, liver disease, biomarker, diagnosis, monitoring

## Abstract

**Simple Summary:**

Proteomics is a discipline investigating the proteins present in a specific biological environment, aiming to understand better the processes that take place in specific districts but also aiming for the possible discovery of new biomarkers. Herein, the fecal proteome of healthy dogs and dogs suffering from different hepatobiliary disorders was investigated. The study highlighted qualitative and quantitative differences between the groups of patients under analysis, leading us in particular to hypothesize a possible role for proteins such as fibronectin, haptoglobin, and trefoil factor 2. The present results need to be further confirmed by other tests and studies, but the direction taken appears promising.

**Abstract:**

In the present study, the fecal proteomes of clinically healthy dogs (HD = n. 10), of dogs showing clinical, ultrasonographic, and/or laboratory evidence of different hepatobiliary dysfunction (DHD = n. 10), and of dogs suffering from chronic hepatitis (CHD = n. 10) were investigated with an Ultimate 3000 nanoUPLC system, coupled to an Orbitrap Fusion Lumos Tribrid mass spectrometer. Fifty-two different proteins of canine origin were identified qualitatively in the three study groups, and quantitative differences were found in 55 proteins when comparing groups. Quantitatively, a total of 41 and 36 proteins were found differentially abundant in the DHD and CHD groups compared to the control HD, and 38 proteins resulted dysregulated in the CHD group as compared to the DHD group. Among the various proteins, differently abundant fecal fibronectin and haptoglobin were more present in the feces of healthy and DHD dogs than in chronic ones, leading us to hypothesize its possible diagnostic/monitoring role in canine chronic hepatitis. On the other hand, the trefoil factor 2 was increased in DHD dogs. Our results show that the analysis of the fecal proteome is a very promising field of study, and in the case of dogs suffering from different hepatobiliary disorders, it was able to highlight both qualitative and quantitative differences among the three groups included. Results need to be confirmed with western blotting and in further studies.

## 1. Introduction

The study of the proteome in a specific substrate is called proteomics. This discipline is used to understand better the processes that take place in specific biological environments but also aims at the possible discovery of new biomarkers to differentiate a healthy status from a disease status and to monitor disease/treatment response [1]. This approach has recently been applied to fecal samples and intestinal biopsies in healthy and diseased animals to investigate and better understand the canine and feline gastrointestinal (GI) tract and related digestive disorders [2,3,4,5,6,7,8]. The study/discovery of new biomarkers in canine and feline GI disorders is indeed a topic of great interest [9].

In recent years, numerous studies have characterized the so-called intestine–liver axis, which indicates the mutual interaction between the intestine/intestinal microbiota and the liver [10]. However, almost all these studies tend to emphasize how changes in the intestinal environment cause liver function alterations thanks to the mediation of portal circulation. It thus clearly emerges that a primitive alteration of intestinal barrier permeability, typical of the “leaky gut”, negatively influences liver function by increasing the arrival of bacterial metabolites and live or dead bacteria to the liver, which can increase levels of hepatocellular apoptosis, reduce the hepatic detoxifying power, and stimulate liver inflammation [10]. On the other hand, the modulation action of the intestinal microbiota and the enteric environment in the course of pathologies that arise primarily in the liver and alter the mechanism of bile and antibody secretion towards the intestine is much less studied. Hepatic bile acids and antibody secretion are crucial in the modeling of intestinal microbial communities by the liver. Recent studies show how hepatic steatosis of a toxic, hypoxic, or alimentary nature can induce alterations of the intestinal microbiota through a modified secretion of primary bile acids and altered reabsorption of the secondary ones [11]. Liver cirrhosis and hepatic failure due to a portosystemic shunt are conditions associated with profound alterations of the intestinal microbiota and damage to the intestinal barrier, resulting in direct modifications of the epithelial, vascular, and immune functions [11]. Small bowel dysmotility is an intestinal alteration typically related to primary liver injury [12]. In human medicine, it has been verified that the alteration of intestinal transit, with greater emphasis on slowing down the duodenal one, is directly proportional to the extent of primary liver damage [13]. This slowdown appears, in turn, to be closely correlated with the overgrowth of small intestinal bacteria, which is, in turn, correlated with the onset of abdominal pain, diarrhea, and systemic endotoxemia [12,14]. A slowdown in hepatic blood flow, generating portal hypertension, also leads to increased intestinal transudation, oxygen reduction in the intestinal environment, and structural and functional changes of the intestinal mucosa, with increased intestinal permeability and translocation of bacteria and metabolites [14]. In these conditions, the fecal proteome is altered, indicating peculiar modifications that can have an important diagnostic interest. The fecal host proteome includes proteins primarily secreted from the gastrointestinal tract (i.e., enzymes, but also mucus, secretory and immune proteins, and proteins resulting from intestinal mucosal cell turnover) [15,16]. Furthermore, the fecal proteome contains proteins that derive from the microbiota and its metabolism [17] which can change, as previously said, following an alteration of the intestinal environment (e.g., reduction in motility and bacterial overgrowth) due to impaired liver function. We further hypothesized that the primary metabolic function of the liver may influence the presence of canine (self) protein in feces. The fecal proteome is, therefore, intrinsically complex because it contains groups of proteins deriving (a) from the host, (b) from the microbiota, and (c) from the remains of the dietary proteome after the metabolic action of the liver [18,19,20].

Given the central role of the liver in the potential transformation of all three of these protein fractions, the aim of this study was to evaluate, for the first time in dogs, possible changes occurring in the GI tract during hepatobiliary disorders and their comparison with controls through the analysis of the fecal proteome, focusing on proteins of canine origin.

## 2. Materials and Methods

### 2.1. Patients

In the present study, three groups of 10 dogs each were enrolled at the Veterinary Teaching Hospitals, Universities of Camerino and Pisa. The first group consisted of clinically healthy dogs (HD) with a history negative for previous diseases, the second of dogs (DHD) showing clinical, ultrasonographic, and/or laboratory evidence of different hepatobiliary dysfunction, and the third of dogs (CHD) suffering specifically from histologically diagnosed chronic hepatitis [21]. To be included in the study, in all cases, no episodes of diarrhea were to be reported in the last month preceding the inclusion (only for groups HD and DHD), and all patients had to be provided with a negative routine copromicroscopic evaluation (i.e., flotation), including a rapid test for *Giardia*, at the time of enrollment or within the previous 15 days, or be provided with a history of regular deworming. For almost all patients of groups DHD and CHD, the results of the haemato-biochemical evaluations carried out at the time of inclusion or performed at least in the previous three months for diagnostic purposes or periodic/planned monitoring evaluations were available. In group DHD, other diagnostic investigations, such as abdominal ultrasonography or hepatic fine needle aspirates, were performed when deemed necessary to achieve a diagnosis and properly manage the patient; in other cases, they were already available as recently made.

No sampling/exam, except fecal proteome evaluations, was performed for the exclusive purpose of the present study but rather in the interest of the patients for diagnostic/clinical aims related to their clinical conditions, as previously reported. The owners of all dogs included signed an informed consent, and the study was conducted in agreement with DL n.26/04-03-2014 (Italian Law) implementing the EU/Directive 2010/63.

### 2.2. Samplings and Protein Extraction, Digestion, and Purification

Naturally voided fecal samples, frozen at −20 °C immediately after evacuation, were used in this study. From each sample belonging to each study group (HD, DHD, CHD), 2 g of stools were collected and pooled (20 g of feces for each group), and all subsequent steps were performed on ice. Each pool from each study group was separately resuspended in three volumes (60 mL) of phosphate buffer saline (PBS) containing a 1:100 diluted protease inhibitor cocktail (Sigma-Aldrich, Saint Louis, MO, USA): this solution was shacked with a magnetic stirrer for one hour on ice and subsequently centrifuged at 10,000× *g* for 20 min. The resulting solutions were subjected to three filtration steps using a filter paper (three times) first, followed by 0.45 μm (one time) and 0.20 μm (one time) sterile membranes (Whatman, Maidstone, UK). To each filtered solution, 90% ammonium sulfate was slowly added, maintaining the samples on ice and in agitation for 30 min. Finally, these solutions were centrifuged at 27,000× *g* for 30 min. After centrifugation, the supernatant was removed, the precipitate was resuspended in 1 mL of PBS buffer, and the total protein content was determined according to the Bradford method [22]. The EasyPep™ Mini MS Sample Prep Kit (Thermo Fisher Scientific, Rockford, IL, USA) was used according to the manufacturer’s instructions for protein extraction, digestion, and purification. At the end of the protein extraction procedure, each sample was dried using a vacuum centrifuge concentrator, and each pellet containing 0.1 mg of proteins was resuspended in 100 μL of 0.1% formic acid for the subsequent LC-MS analysis.

### 2.3. LC-MS/MS Analysis

Analyses were carried out on an Ultimate 3000 nanoUPLC system (Thermo Fisher Scientific), coupled to an Orbitrap Fusion Lumos Tribrid mass spectrometer (Thermo Fisher Scientific, Waltham, MA, USA). Peptide separations were performed using an Easy-Spray PepMap RSLC C18 column, (2 µm, 500 × 0.075 mm, Thermo Fisher Scientific). A sample volume of 1 μL was injected. The samples were eluted using a 120 min gradient at a flow rate of 300 nL/min, (4% to 40% solvent B for 90 min, 40% to 50% for 5 min, 50% to 90% for 5 min, hold at 90% for 5 min, and 105–120 min hold at 5%). The composition of the mobile phase was as follows: formic acid at 0.1% (solvent A) and formic acid solution at 0.1%/Acetonitrile (20%/80%, solvent B). ESI ion source operated in a positive ionization mode (1500 V), and the capillary temperature was at 275 °C. MS1 spectra were acquired using the Orbitrap analyzer operating in data-dependent acquisition mode, ranging from 375 *m*/*z* to 1500 *m*/*z*, at a resolution of 120,000 (at 200 *m*/*z*), with standard automated gain control (AGC, a maximum injection time of 50 ms and charge state of 2–5). For MS2 analysis, precursors were isolated in a 2.0 Da window and fragmented in HCD mode using a normalized collision energy of 30% and selected based on their intensity from all signals (charge state from 2+ to 5+). To prevent repeated fragmentation of the same peptide ion, dynamic exclusion was set to 30 s. The MS2 spectra were acquired using the Orbitrap analyzer at a resolution of 50,000 (at 200 *m*/*z*). For each condition, the experiments were performed in quadruplicate.

### 2.4. Data Analysis

Protein identification and label-free quantification (LFQ) were performed using the Proteome Discoverer (v2.5) (Thermo Fisher Scientific, Bremen, Germany). Identification was conducted using the Sequest HT search engine (Thermo Fisher Scientific, Bremen, Germany) and the following parameters: stable modification carbamidomethyl (C), oxidation (M) and acetylation (protein N terminus) as variable modifications, the Uniprot database (*Canis lupus familiaris* entries), trypsin as a specific protease, and a maximum of two missed cleavages, including common contaminants. Mass accuracy was set to 4.5 ppm for precursor ions and 0.5 Da for Orbitrap MS/MS data. A minimum peptide length of five amino acids was accepted. The “match between runs” option enabled the transfer of identifications across samples within a time window of 2 min of the aligned retention times. The confidence level for peptide identifications was estimated using the Percolator algorithm with decoy database searching. Identifications were filtered by false discovery rate (FDR) validation based on a q value set to 0.01. The LFQ of identified proteins, referred to as unique peptides, required a minimum ratio count of two and was calculated based on the raw spectral protein intensity. For each condition, raw intensities were logarithmized, normalized to the calculated average, and used for downstream analyses. Student’s *t*-test was performed on the normalized protein intensities, and proteins with *p* < 0.05 and a fold change > 1.5 were considered significantly altered in abundance between the samples.

### 2.5. Bioinformatics Analysis of Omics Data 

Bioinformatic analysis of the proteomic data was performed as previously described [23]. Briefly, to characterize the function of proteins, gene ontology (GO) annotation and KEGG pathways enrichment analyses, together with an investigation of protein–protein interaction networks, were performed using STRING v.11.5 (https://string-db.org/ accessed on 18 May 2023). The differentially abundant proteins were analyzed for candidate functions and pathways enrichment, setting *Canis lupus familiaris* as taxonomy, *p* < 0.05, and gene count > 2 as the cut-off point. We also retrieved known interactions experimentally determined (pink edge) and on a curated database (light blue edge), excluding all other prediction methods implemented in STRING (such as co-expression and text-mining). Additional white nodes and network depth were kept to the minimum value to exclude as many false positive interactions as possible. 

## 3. Results

The patients included in the study are reported in Table 1. In the DHD group, patients were all showing different clinical and/or laboratory impairment of hepatic function and/or ultrasonographic signs of liver suffering, such as hepatic enlargement and/or inhomogeneity of the parenchyma and/or altered echogenicity and/or suspended material or stones in the gallbladder. In these dogs, a nonspecific diagnosis of hepatobiliary disease in association or not with other pathologic conditions was made. In the third group (CHD), all dogs variably showed altered laboratory and ultrasonographic hepatobiliary findings, and in all cases, a histologic diagnosis of chronic hepatitis was made.

**Figure 1:**
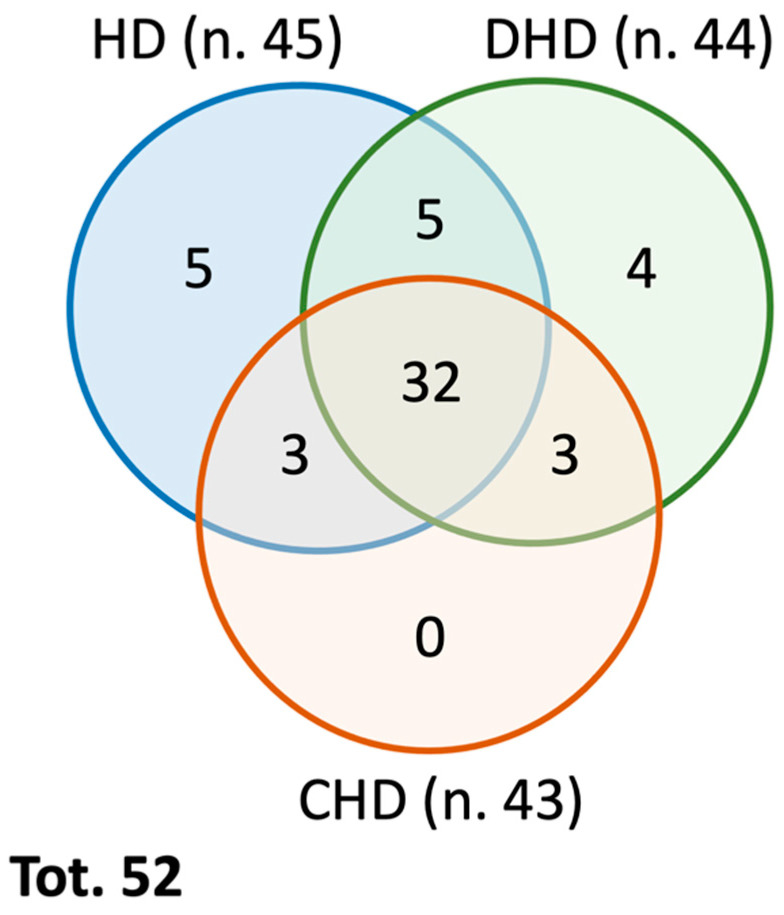
Venn Diagram summarizing the qualitative distribution of the identified proteins in the different study groups (as reported in Table 2).

From a qualitative analysis of the proteomics data, it is possible to underline that, in total, 79 different proteins of canine origin were identified in the three study groups, in particular, 66 in the HD group, 61 in the DHD group, and 53 in the CHD group. After applying proper filters, i.e., eliminating proteins identified by a single peptide, of the proteins with a false discovery rate (FDR) confidence other than high (i.e., medium or low), and the proteins not detected in all four technical replicates performed, 52 different total proteins remained: 45 in the HD group, 44 in the DHD group, and 43 in the CHD group (Figure 1). A total of 32 same proteins were found in all three groups, while 20 were differently present within the three groups; 5 proteins were found in the HD and DHD groups but not in the CHD, 3 proteins were found in the HD and CHD groups but not in the DHD, 3 proteins were found in the DHD and CHD groups but not in the HD. Furthermore, 5 proteins were found only in the HD group, 4 proteins were found only in the DHD group, and no proteins were found only in the CHD group (Figure 1). All qualitative results are reported in Table 2.

**Figure 2:**
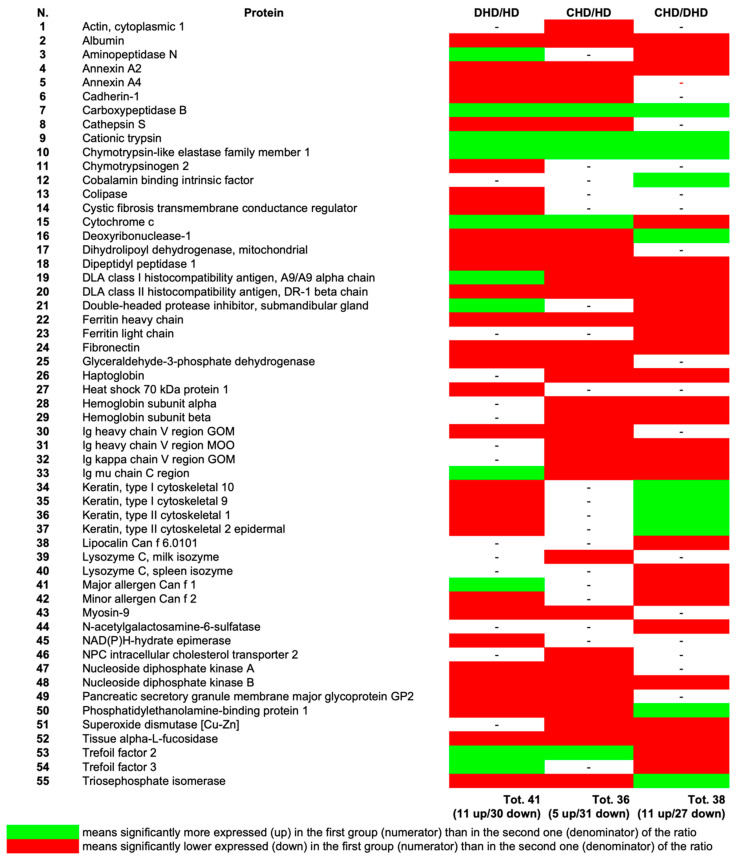
Quantitative analysis of the proteins present in the 3 study groups; abundance ratio for couples of groups. The figure shows only the proteins with statistically significant differences in at least one of the ratios (DHD/HD; CHD/HD; CHD/DHD). *p* < 0.05. (See Supplementary Tables S1–S3 for protein modulations).

Furthermore, we also conducted a label-free quantitative proteomics analysis to study alterations in protein abundances and sought possible evidence for and understanding of the molecular mechanisms involved in DHD and CHD. Differential protein abundance was significant when the abundance increased or decreased with a fold change of 1.5 and a *p*-value < 0.05. A total of 41 and 36 proteins were found differentially abundant in the DHD and CHD groups compared to the control HD (Figure 2). In addition, 38 proteins were dysregulated in CHD as compared to the DHD group (Figure 2). 

**Figure 3:**
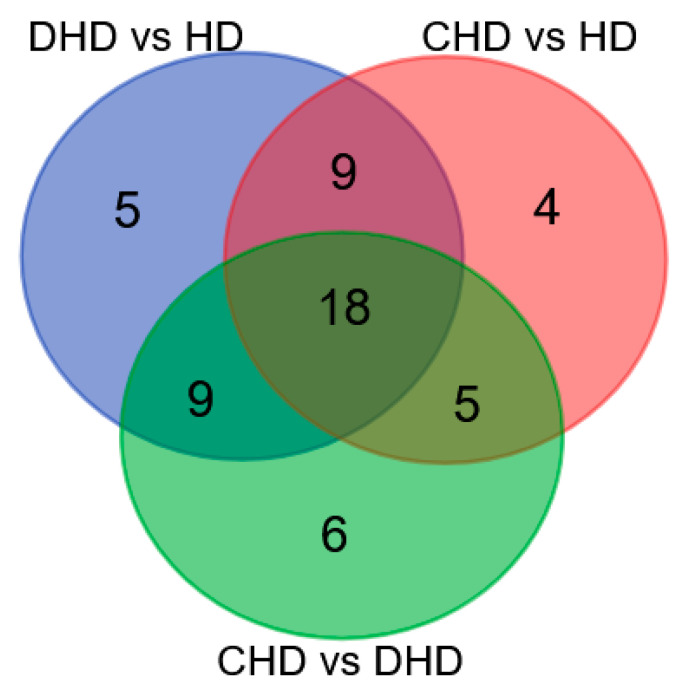
Venn Diagram summarizing dysregulated proteins detected across all the samples (see Supplementary Tables S1–S3 for gene/protein name).

Proteomic results demonstrated that there were 18 common proteins modulated in the three comparisons. There were also five proteins that were found to be dysregulated exclusively in DHD (i.e., Colipase, NAD(P)H-hydrate epimerase, cystic fibrosis transmembrane conductance regulator, Heat shock 70 kDa protein 1, and Chymotrypsinogen 2), and only four in CHD (i.e., hemoglobin subunit alpha, NPC intracellular cholesterol transporter 2, Actin, cytoplasmic 1, and lysozyme C, milk isozyme) as compared to the HD group. Interestingly, six proteins were found to have different abundances between CHD and DHD groups (i.e., Lipocalin Can f 6.0101, Ferritin light chain, cobalamin binding intrinsic factor, N-acetylgalactosamine-6-sulfatase, hemoglobin subunit alpha, and lysozyme C, spleen isozyme). Overlapping and non-overlapping dysregulated proteins are shown in Figure 3.

**Figure 4:**
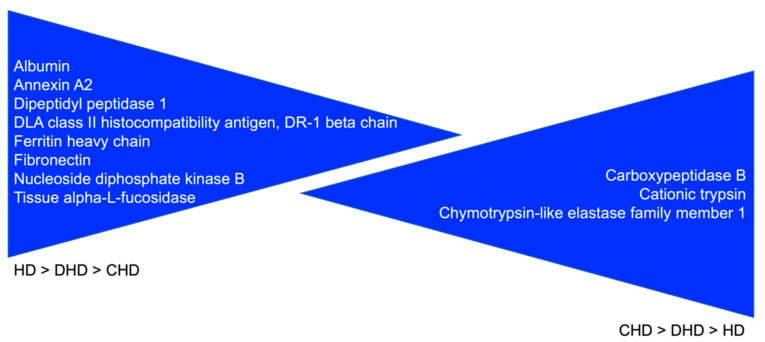
Proteins with a clear specific trend: HD > DHD > CHD and CHD > DHD > HD.

Proteins progressively significantly decreasing from HD to DHD until CHD (from a healthy condition to the most severe state of disease—see Table 1) and from CHD to DHD until HD (from the most severe state of disease to a healthy condition—see Table 1) are reported in Figure 4.

To reveal signaling pathways and interaction networks perturbed by the disease state, we performed a bioinformatics analysis on the proteins whose abundance significantly changed. Enrichment analysis detected enriched cellular component GO terms and KEGG pathways. In particular, proteomic results revealed that dysregulated proteins of DHD and CHD were mainly proteins of the extracellular region, whereas the proteins that differentiate DHD from CHD additionally include proteins belonging to the hemoglobin complex. 

Statistically significant enriched KEGG pathways (*p*-value < 0.05) were detected for proteins dysregulated in CHD as compared to HD, and they included lysosome, apoptosis, viral myocarditis, and bacterial invasion of epithelial cells pathways (Supplementary Table S4). 

**Figure 5:**
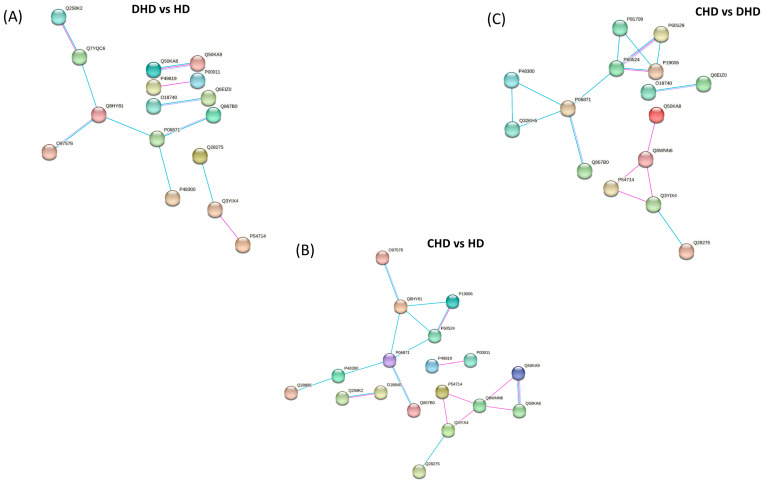
Protein network of modulated proteins in (**A**) DHD vs. HD, (**B**) CHD vs. HD, and (**C**) CHD vs. DHD by using STRING. The minimum required interaction score was set as the medium confidence (0.400) and the disconnected nodes in the network were hidden. Pink edges represent interactions experimentally determined, while light blue edges represent known. Proteins are indicated by their Uniprot accession number.

To determine the key proteins in the function network, the STRING online tool was used to analyze the protein–protein interaction (PPI) networks. The analysis resulted in three PPI networks (shown in Figure 5) with an average local clustering coefficient (i.e., indication of the embeddedness of single nodes) and an average node degree (i.e., number of interactions, at the score threshold, that a protein has on the average in the network) of 0.375 and 0.688, 0.494 and 1.29, and 0.408 and 1.1. 

## 4. Discussion

The present study investigated for the first time the fecal proteome of dogs suffering from different hepatobiliary disorders, and its comparison with controls. Interestingly, many proteins were both qualitatively and quantitively differently abundant in the three groups of patients included in this study.

A bioinformatic evaluation of the pathways in which the dysregulated proteins play a role was also performed. The identified dysregulated proteins detected for CHD were implicated in lysosome pathways [Dipeptidyl peptidase 1 (CTSC), Cathepsin S (CTSS), Tissue alpha-L-fucosidase (FUCA1), and NPC intracellular cholesterol transporter 2 (NPC2) proteins]; apoptosis [Cytochrome c (CYCS), Dipeptidyl peptidase 1 (CTSC), Cathepsin S (CTSS), Actin, cytoplasmic 1 (ACTB); Cytochrome c 320 (CYCS), Dipeptidyl peptidase 1 (CTSC), Cathepsin S (CTSS), Actin, cytoplasmic 1 (ACTB)]; viral myocarditis [DLA class I histocompatibility antigen, A9/A9 alpha chain (DLA88), Cytochrome c (CYCS), Actin, cytoplasmic 1 (ACTB)]; and bacterial invasion of the epithelial cells [and Actin, cytoplasmic 1 (ACTB), Cadherin-1, Fibronectin (FN1)]. The protein–protein interaction analysis showed that dysregulated identified proteins have more interactions among themselves than expected for a random set of proteins of the same size and for the degree of distribution drawn from the genome. The medium-low average local clustering coefficient suggests the presence of little connected neighborhoods in the network, as indicated by the presence of small groups of interacting proteins. Such an enrichment demonstrates that the identified proteins are at least partially biologically connected.

Regarding those proteins (n. 5) qualitatively present only in the HD and DHD groups (Table 2), the Ferritin-heavy chain responsible for iron storage and presenting ferroxidase activity [24], as well as hemoglobin subunits alpha and beta, are findings difficult to justify. However, the first one is potentially interesting as it was found to be significantly less abundant in the DHD and CHD groups than HD, and less abundant in CHD than in DHD samples. Similarly, Fibronectin, a protein primarily involved in numerous biological processes, including developing and maintaining cellular organization, was more represented in HD than in DHD and CHD (Figure 2 and Figure 4). This protein was interestingly found to be increased in the liver of dogs naturally infected with *Leishmania* spp. as a result of increased fibrogenesis [25], and therefore its low abundance in CHD dogs could be attributable to its increased intrahepatic deposition, which could occur progressively and be greater in the most severe conditions. Another aspect that is also extremely interesting, but for a different reason, is the downregulation of haptoglobin in CHD, which is produced by the liver and whose most important function is to recycle heme iron in the liver by combining itself with free plasma hemoglobin, which in addition to being a positive acute phase protein (increasing in case of inflammation) was found to be markedly decreased in canine late stage cirrhosis [26], aspects that would seem to justify our findings.

Proteins qualitatively found in HD and CHD groups (n. 3) and not in the DHD group (Table 2) were all proteins with general nonspecific functions (as far as the purpose of this study is concerned) [27,28]. For this reason, the interesting finding of Nucleoside diphosphate kinase B quantitatively significantly decreasing in the three groups, from HD to CHD, cannot be contextualized.

Within those proteins more abundant in DHD and CHD than HD, Chymotrypsin-like elastase has been associated with exocrine pancreatic insufficiency in man [29]; therefore, it is difficult to contextualize herein. However, it is interesting to underline that this protein was significantly more present in the CHD group than in DHD. The second one, Cubilin, is a fundamental component of the membrane-bound receptor necessary for the uptake of intestinal cobalamin, and it is worth noting that degenerative liver disease was associated with genetically confirmed hereditary cobalamin malabsorption in two dogs as well as in other species presenting hypocobalaminemia [30]. We would have expected a deficiency of this protein in DHD and especially CHD patients and not in HD; however, even if it is difficult to interpret in our context, or probably for this reason, we believe this finding is remarkable. Finally, trefoil factor 2 is a peptide secreted by goblet cells playing an important role in gastrointestinal healing after epithelial injury and, in dogs, it seems to be expressed exclusively in the stomach [31]. However, it is overexpressed in human medicine in different cancers, including gastric and cholangiocarcinoma [32,33], and in the hepatobiliary mucosa associated with hepatolithiasis [34]. This partially justifies its presence in our samples, and it is interesting to highlight that it is more abundant in DHD and CHD as compared to HD, and it is also significantly more present in the DHD compared to CHD, possibly as a consequence of its production only in diseased dogs (and none in healthy dogs), with a higher production and release in more “active” conditions, possibly also involving bile ducts (DHD) in comparison to chronic conditions (CHD).

Then, analyzing proteins that were less abundant in DHD and CHD, Annexins A2 and A4 are both calcium-dependent phospholipid-binding proteins involved in exocytosis and are upregulated in different neoplasms, including hepatocellular carcinoma and liver fibrosis [35,36,37,38]. Interestingly, Annexin A4 is expressed only by epithelial cells and encodes a protein that has possible interactions with ATP, has anticoagulant activity in vitro, and also inhibits the activity of phospholipase A2, having a protective effect against inflammation [39]. This protein regulates cell growth and apoptosis, modulating membrane permeability and actively participating in its repair when altered. Annexin A4, by binding Ca^2+^ and phospholipids, can rapidly repair small micron-sized holes in the membrane. It is not surprising that such a protein is present in the feces of healthy dogs, as it is strongly recruited and implicated in the prevention of apoptosis phenomena, although it is not known the reason for its absence in those of DHD and CHD patients. It is, however, conceivable that in both categories of dogs, an increase in enterocytes damage and apoptosis, linked to an altered entero-hepatic recirculation of some potentially toxic metabolites (i.e., secondary bile acids) caused by a modified hepatocellular activity, could increase the recruitment of these proteins at the level of the enteric mucosa [40]. Remarkably, and in accordance with the above, annexin A2 was found quantitatively significantly decreasing in the three groups, from HD to CHD. No specific correlation with the aim of this study was found for the three other proteins found only in the HD group.

Finally, among the proteins less abundant in DHD as compared to HD, the cystic fibrosis transmembrane conductance regulator is the most interesting one for the patients included herein, being that its defective production causes the human genetic disease cystic fibrosis by reducing the permeability of Cl^-^ in many tissues, which in turn impairs salt absorption and fluid secretion [41]. Its altered production is also involved in the determinism of many hepatobiliary conditions collectively referred to as cystic fibrosis liver disease, representing one of the main causes of mortality in cystic fibrosis human cases [42]. Therefore, also in this case, as in others previously, it appears interesting that the protein is present in sick and not in healthy dogs, but above all that, although it is typically associated with hepatic fibrosis/cirrhosis in humans, it is not present in our CHD group. Although it could be hypothesized that its absence in CHD dogs is due to its impaired production, this hypothesis does not explain its absence in healthy (HD) ones.

As for the results obtained with the present study, some other proteins showed an interesting and statistically significant trend, progressively decreasing from HD to DHD until CHD (from the healthy condition to the most severe state of disease—see Table 1) as well as from CHD to DHD until HD (from the most severe state of disease to the healthy condition—see Table 1) (Figure 4). Among these proteins, in addition to those already discussed above, of higher interest appears Albumin (Figure 4), higher in healthy dogs than in diseased ones. Although mean serum albumin was normal in the DHD and CHD groups (Table 1), it is interesting that it has been seen to be significantly decreased in the feces of diseased dogs, with the lowest levels in chronic patients. Such condition led us to reasonably hypothesize, in a completely speculative way, that the reduction in fecal albumins could represent a marker of chronic liver disease earlier than its serum value. Similarly, Dipeptidyl peptidase 1 was also found to decrease from healthy to disease status. It is a lysosomal cysteine protease involved in the processing of neutral serine proteases (granzymes A and B), which are expressed granules of activated cytotoxic lymphocytes. Once activated, granzymes A and B are required for cytotoxic lymphocyte granule-mediated apoptosis [43]. No link was found between this protein and canine hepatobiliary disorders, but interestingly, another isoform, Dipeptidyl peptidase 4, was associated in men with hepatic steatosis and various chronic liver diseases [44]. Therefore, its reduced presence in CHD dogs’ feces could be due to its increased deposition in the liver.

If comparing the present results with the previous literature on the canine fecal proteome, it is confirmed that more work is needed on this topic, considering that a different methodology was used in the present study. For example, Superoxide dismutase [Cu-Zn], a protein found downregulated in CHD as compared to HD or DHD in our study, was also found in dogs naturally infected with *Trichuris vulpis* (Congress Proceedings) [6] and suffering from chronic enteropathy [7]; however, it was variably detected in two different groups of healthy dogs (in one group yes [7] and in one no [3]). Similarly, different Myosin isoforms were found in parasitized dogs [6] and in our study’s HD dogs but not in healthy dogs of the literature [3], making it difficult to compare studies using different techniques. Again, always considering this premise, we can read the finding of a Chymotrypsin-like elastase family member 1 in the two groups of diseased dogs, but not in healthy ones, of the present study, while a similar protein, Chymotrypsin-C-like, was detected in healthy and acute diarrheic dogs, as well as in dogs suffering from intestinal lymphangiectasia [3,5,8].

One limitation of this study is that it was impossible to fully comply with one of the inclusion criteria (absence of diarrhea in the previous month) in the CHD group, as some sporadic episodes were reported as understandably expected, given the underlying disease. Additionally, a possible limitation is the absence of the exact cyto/histological characterization of patients included in the DHD group. Moreover, but beyond the objectives of the present study as it was designed, the proteins hypothesized as potential biomarkers must be identified by western blotting analysis.

## 5. Conclusions

In conclusion, the present first fecal proteomic evaluation performed in dogs suffering from different hepatobiliary disorders highlights qualitative and quantitative differences among the three groups included. Fecal fibronectin and haptoglobin were more present in the feces of healthy and DHD dogs than in chronic ones, leading us to hypothesize its possible diagnostic/monitoring role, to be confirmed with western blotting and in further studies in canine chronic hepatitis. On the other hand, the increased presence of trefoil factor 2 in dogs with evidence of different hepatobiliary disorders may suggest a potential role for this protein in such conditions. Further studies are warranted in this direction.

## Figures and Tables

**Table 1:** Signalment of patients included in the study and the associated diagnosis with division into groups.

	Breed	Age (years)	Sex	Hepatobiliary Enzymes (MEAN Values per Group)	Associated Conditions
**Healthy dogs** **(HD group)**	American Bulldog	3	F	/	/
	American Bulldog	2	F		
	American Bulldog	4	F		
	Belgian Shepherd (Malinois)	4+½	F		
	Border Collie	6	F		
	English Setter	10	M		
	German Hound	7	M		
	Mestizo	12	F		
	Whippet	4	F		
	Whippet	6	F		
**Dogs with evidence of different hepatobiliary disorders (DHD group)**	Mestizo	14	F	GLU *: 140.78 (r.i.: 65.00–118.00 mg/dL) AST **: 75.95 (r.i.: 23.00–66.00 UI/L) ALT *: 181.42 (r.i.: 21.00–102.00 UI/L) GGT *: 7.67 (r.i.: 1.20–6.40 UI/L) CHOL *: 231.85 (r.i.: 135.00–270.00 mg/dL) ALP *: 687.11 (r.i.: 20.00–156.00 UI/L) TRI **: 89.75 (r.i.: 20.00–112.00 mg/dL) ALB *: 3,19 (r.i.: 2.60–3.30 g/dL) Bil-Tot **: 0.75 (r.i.: 0.10–0.50 mg/dL)	/
	Pinscher	12	F		Mitral insufficiency (cardiac)
	Mestizo	2	M		Polytrauma
	Dachshund	11	M		Diabetes mellitus
	English Cocker Spaniel	10	M		Hepatocutaneous syndrome
	Golden Retriever	6	F		/
	Mestizo	9	M		Cutaneous mastocytoma and bladder cancer
	Mestizo	10	M		Bite injury
	Akita	9	F		/
	Chihuahua	5	M		Epilepsy
**Dogs suffering from chronic hepatitis (CHD group)**	Mestizo	9	F	GLU: 103.20 (r.i.: 80.00–125.00 mg/dL), AST **: 148.88 (r.i.: 15.00–40.00 U/L), ALT: 405.7 (r.i.: 20.00–70.00 U/L), GGT: 11.12 (r.i.: 2.00–11.00 U/L), CHOL: 230.3 (r.i.: 120.00–280.00 mg/dL), ALP: 1662.3 (r.i.: 45.00–250.00 U/L), ALB: 3.67 (r.i.: 2.60–4.10 g/dL), TRI *: 79.33 (r.i.: 25.00–90.00 mg/dL), Bil-Tot: 0.46 (r.i.: 0.00–0.30 mg/dL)	/
	Pinscher	10	M		
	Mestizo	8	F		
	West highland White Terrier	10	F		
	Mestizo	2	F		
	Cavalier King Charles Spaniel	6	F		
	French Bulldog	3	F		
	Golden Retriever	3	M		
	Mestizo	10	F		
	Mestizo	9	M		

**GLU**: Glucose; **ALT**: Alanine aminotransferase; **AST**: Aspartate aminotransferase; **GGT**: Gamma-glutamyl transferase; **CHOL**: Cholesterol; **ALP**: Alkaline phosphatase; **ALB**: Albumin; **TRI**: Triglycerides; **Bil-Tot**: Bilirubin, total. **r.i.**: reference intervals. Underlined the values outside the reference intervals. * data available in 9/10 patients. ** data available in 8/10 patients.

**Table 2:** Qualitative analysis of the proteins present in the three study groups.

N.	Protein Name [*OS* = *Canis lupus familiaris*]	HD (Coverage %)	DHD (Coverage %)	CHD (Coverage %)	Accession	MW [kDa]	calc. pI
1	Actin, cytoplasmic 1	X (57)	X (23)	X (21)	O18840	41.7	5.48
2	Albumin	X (69)	X (72)	X (56)	P49822	68.6	5.69
3	Aminopeptidase N	X (54)	X (56)	X (54)	P79143	110.2	6.18
4	Anionic trypsin	X (60)	X (60)	X (60)	P06872	26.4	4.83
5	**Annexin A2** ^[d]^	X (26)	-	-	Q6TEQ7	38.6	7.31
6	**Annexin A4** ^[d]^	X (10)	-	-	P50994	35.8	5.92
7	Cadherin-1	X (25)	X (25)	X (25)	F1PAA9	97.7	4.81
8	Carboxypeptidase B	X (28)	X (44)	X (44)	P55261	47.6	6.6
9	Cationic trypsin	X (81)	X (81)	X (81)	P06871	26.2	8.07
10	**Chymotrypsin-like elastase family member 1** ^[c]^	-	X (78)	X (77)	Q867B0	27.9	8.46
11	Chymotrypsinogen 2	X (58)	X 46)	X (35)	P04813	27.8	7.2
12	Cobalamin binding intrinsic factor	X (9)	X (34)	X (36)	Q5XWD5	45	5.78
13	Colipase	X (14)	X (31)	X (31)	P19090	12	8.02
14	**Cubilin** ^[c]^	-	X (2)	X (3)	Q9TU53	397.2	5.44
15	**Cystic fibrosis transmembrane conductance regulator** ^[e]^	-	X (1)	-	Q5U820	168.4	8.9
16	Cytochrome c	X (49)	X (64)	X (55)	P00011	11.6	9.58
17	Deoxyribonuclease-1	X (61)	X (9)	X (64)	Q767J3	31.5	5.45
18	Dihydrolipoyl dehydrogenase, mitochondrial	X (35)	X (9)	X (27)	P49819	54.1	7.84
19	Dipeptidyl peptidase 1	X (48)	X (41)	X (51)	O97578	49.4	7.03
20	DLA class I histocompatibility antigen, A9/A9 alpha chain	X (13)	X (18)	X (19)	P18466	40.4	5.87
21	DLA class II histocompatibility antigen, DR-1 beta chain	X (15)	X (22)	X (19)	P18470	30.1	6.15
22	Double-headed protease inhibitor, submandibular gland	X (23)	X (30)	X (17)	P01002	12.8	7.93
23	**Ferritin heavy chain** ^[a]^	X (15)	X (17)	-	Q95MP7	21.3	5.88
24	**Ferritin light chain** ^[e]^	-	X (27)	-	Q53VB8	20.1	6
25	**Fibronectin** ^[a]^	X (4)	X (7)	-	Q28275	243.1	5.99
26	Glyceraldehyde-3-phosphate dehydrogenase	X (24)	X (9)	X (16)	Q28259	35.8	8.12
27	**Haptoglobin** ^[a]^	X (13)	X (44)	-	P19006	36.4	6.09
28	**Hemoglobin subunit alpha** ^[a]^ *	X (27)	X (30)	-	P60530 HD P60529 DHD	15.4 HD 15.2 DHD	8.06
29	**Hemoglobin subunit beta** ^[a]^	X (42)	X (64)	-	P60524	16	8.05
30	Ig heavy chain V region GOM	X (26)	X (26)	X (26)	P01784	12.4	5.4
31	Ig heavy chain V region MOO	X (42)	X (39)	X (26)	P01785	12.7	4.72
32	**Ig kappa chain V region GOM** ^[b]^	X (34)	-	X (34)	P01618	12	6.61
33	Ig mu chain C region	X (8)	X (8)	X (8)	P01874	48.9	6.13
34	Keratin, type I cytoskeletal 10	X (27)	X (22)	X (27)	Q6EIZ0	57.7	5.15
35	**Keratin, type I cytoskeletal 9** ^[b]^	X (4)	-	X (6)	O18740	76.3	5.95
36	Keratin, type II cytoskeletal 1	X (20)	X (21)	X (18)	Q6EIY9	63.8	7.84
37	Keratin, type II cytoskeletal 2 epidermal	X (12)	X (14)	X (14)	Q6EIZ1	64.5	7.74
38	Lysozyme C, milk isozyme	X (33)	X (74)	X (46)	P81708	14.5	8.29
39	**Lysozyme C, spleen isozyme** ^[e]^	-	X (48)	-	P81709	14.6	8.81
40	**Minor allergen Can f 2** ^[e]^	-	X (38)	-	O18874	20.2	5.02
41	**Myosin-9** ^[d]^	X (2)	-	-	Q258K2	226.3	5.6
42	NPC intracellular cholesterol transporter 2	X (42)	X (66)	X (42)	Q28895	16	8.02
43	**Nucleoside diphosphate kinase A** ^[d]^	X (49)	-	-	Q50KA9	17.2	6.01
44	**Nucleoside diphosphate kinase B** ^[b]^	X (64)	-	X (14)	Q50KA8	17.4	7.99
45	Pancreatic secretory granule membrane major glycoprotein GP2	X (13)	X (37)	X (13)	P25291	56.7	5.62
46	**Phosphatidylethanolamine-binding protein 1** ^[d]^	X (67)	-	-	Q3YIX4	20.9	7.49
47	Superoxide dismutase [Cu-Zn]	X (46)	X (46)	X (46)	Q8WNN6	15.9	6.11
48	Tissue alpha-L-fucosidase	X (50)	X (33)	X (17)	P48300	53.7	6.74
49	**Trefoil factor 2** ^[c]^	-	X (69)	X (39)	Q863J2	14.1	7.65
50	Trefoil factor 3	X (54)	X (54)	X (54)	Q863B4	8.9	4.94
51	Triosephosphate isomerase	X (81)	X (37)	X (78)	P54714	26.7	7.33
52	Ubiquitin-60S ribosomal protein L40	X (27)	X (34)	X (34)	P63050	14.7	9.83
		**Tot.45**	**Tot.44**	**Tot.43**			

In **bold** proteins present in 1 and/or 2, but not in all the 3 groups (n. 20).^[a]^ Proteins present in groups HD and DHD (n. 5); ^[b]^ Proteins present in groups HD and CHD (n. 3); ^[c]^ Proteins present in groups DHD and CHD (n. 3); ^[d]^ Proteins present only in HD group (n. 5); ^[e]^ Proteins present only in DHD group (n. 4). * For group HD [*OS = Canis latrans*] while for group DHD [*OS = Canis lupus familiaris*]. The colors are a reference to colors in Figure 1.

## Data Availability

The data presented in this study are contained within the article or Supplementary Material.

## Supplementary Materials

The following are available online at https://www.mdpi.com/article/10.3390/ani13142343/s1, Supplementary Table S1. DHD vs. HD; Supplementary Table S2. CHD vs. HD; Supplementary Table S3. CHD vs. DHD; Supplementary Table S4. Functional enrichment analysis.
