# Supplementary material for: Fecal Proteome Profile in Dogs Suffering from Different Hepatobiliary Disorders and Comparison with Controls
Source: Animals (Basel). 2023 Jul 18;13(14):2343. doi: 10.3390/ani13142343 (PMC10376375; doi:10.3390/ani13142343)
Supplement: Supplementary file 1 [file animals-13-02343-s001.zip › Supplementary Table S4.pdf]

#category	term ID	term description	observed gene count	background gene count	strength	FDR	Matching proteins (labels)
**DHD vs HD**:							
GO Component	GO:0005576	Extracellular region	16	2155	0.67	4.94e-05	DLA88,CLPS,PRSS1,CTSC,ENSCAFP00000009968,ANPEP,CTSS,NAXE,GP2,OBP2B,CELA1,ANXA2,DNASE1,TFF2,CANF2,FN1
**CHD vs HD:**							
GO Component	GO:0005576	Extracellular region	14	2155	0.67	0.00036	DLA88,PRSS1,CTSC,SOD1,LYZF2,CTSS,GP2,HP,CELA1,ANXA2,NPC2,DNASE1,TFF2,FN1
GO Component	GO:0005615	Extracellular space	11	1274	0.79	0.00039	DLA88,PRSS1,CTSC,SOD1,LYZF2,CTSS,GP2,HP,CELA1,ANXA2,TFF2
KEGG	cfa04142	Lysosome	4	155	1.27	0.0155	CTSC,CTSS,FUCA1,NPC2
KEGG	cfa04210	Apoptosis	4	140	1.31	0.0155	CYCS,CTSC,CTSS,ACTB
KEGG	cfa05416	Viral myocarditis	3	79	1.44	0.0228	DLA88,CYCS,ACTG1
KEGG	cfa05100	Bacterial invasion of epithelial cells	3	100	1.34	0.0335	ACTB,CDH1,FN1
**CHD vs DHD:**							
GO Component	GO:0005576	Extracellular region	17	2155	0.74	6.96e-07	DLA88,PRSS1,CTSC,ENSCAFP00000009968,SOD1,CBLIF,ANPEP,HP,OBP2B,CELA1,LYZ,ANXA2,DNASE1,ENSCAFP00000057458,TFF2,CANF2,FN1
GO Component	GO:0005615	Extracellular space	11	1274	0.78	0.00059	DLA88,PRSS1,CTSC,SOD1,CBLIF,ANPEP,HP,CELA1,LYZ,ANXA2,TFF2
GO Component	GO:0005833	Hemoglobin complex	2	5	2.44	0.0209	HBA,HBB
GO Component	GO:0031838	Haptoglobin-hemoglobin complex	2	5	2.44	0.0209	HBA,HBB
KEGG	cfa05143	African trypanosomiasis	3	56	1.57	0.0286	HP,HBA,HBB

Supplementary Table S4. Functional enrichment analysis. Enriched GO component terms and KEGG pathways obtained by String analysis. The table reports the term IDs and description, the observed gene count (which reflects the number of the identified proteins belonging to the enriched GO term/KEGG pathway), the background gene count (i.e. total number of genes related to the indicated GO term/KEGG pathway), the strenght and FDR which indicate the statistical significance of the enrichment, and the name of matching proteins.
